# Togo National Herbarium database

**DOI:** 10.3897/phytokeys.109.25385

**Published:** 2018-09-13

**Authors:** Raoufou Radji, Kossi Adjonou, Marie-Luce Akossiwoa Quashie, Komlan Edjèdu Sodjinou, Francisco Pando, Kouami Kokou

**Affiliations:** 1 Laboratory of Forestry Research, Faculty of Science, University of Lomé, 01BP 1515 Lomé 1, Togo University of Lomé Lomé Togo; 2 Real Jardin Botanico, CSIC, Madrid, Spain Real Jardin Botanico Madrid Spain

**Keywords:** herbarium collection, herbarium sheets, Liliopsida, Magnoliopsida, Occurrence, Plants, RIHA, Togo, University of Lomé, West Africa

## Abstract

This article describes the herbarium database of the University of Lomé. The database provides a good representation of the current knowledge of the flora of Togo. The herbarium of University of Lomé, known also as *Herbarium togoense* is the national herbarium and is registered in Index Herbariorum with the abbreviation TOGO. It contains 15,000 specimens of vascular plants coming mostly from all Togo's ecofloristic regions. Less than one percent of the specimens are from neighbouring countries such as Ghana, Benin and Burkina Faso. Collecting site details are specified in more that 97% of the sheet labels, but only about 50% contain geographic coordinates. Besides being a research resource, the herbarium constitutes an educational collection. The dataset described in this paper is registered with GBIF and accessible at https://www.gbif.org/dataset/b05dd467-aaf8-4c67-843c-27f049057b78. It was developed with the RIHA software (Réseau Informatique des Herbiers d'Afrique). The RIHA system (Chevillotte and Florence 2006, Radji et al. 2009) allows the capture of label data and associated information such as synonyms, vernacular names, taxonomic hierarchy and references.

## Context

Botanical collections are an indispensable tool in the field of biodiversity conservation. Indeed, they provide basic data for the evaluation of species conservation status, a task that presents a significant lack in Africa, especially in Togo (Radji et al. 2010; Billand 2010).

In West Africa as in Central Africa, the level of digital accessible information (DAK) on vascular plants – though far from satisfactory – has been raised by efforts in neighbouring countries (Dauby et al. 2016, Sosef et al. 2017). Remarkable in this regard is the onlined dataset published by Benin (e.g. The census of national forest of Benin (Gaston 2016) and the University of Ghana Herbarium database (Asase 2015). Those contributions spotlighted the lack of information available on plants from Togo, a fact that has been limiting the feasibility of sound floristic and ecological studies in the region until now.

The dataset, here described, narrows this knowledge gap and should be a key resource in the making of a national plant checklist for Togo, monographs, floras and other research crucial to addressing challenges of knowledge, pedagogy, sustainable development and decision-making about natural resources and environments (Chapman 2005; Sousa-Baena et al. 2013).

In a wider context, this dataset contributes to the correction of a global lack of tropical biodiversity data availability (Collen et al. 2008, Feeley and Silman 2011) and to the GBIF Content Mobilization Priorities set in 2017 (https://www.gbif.org/mobilization-priorities-2017).

Data capture of the University of Lomé herbarium collections started in 2003 as part of the RIHA initiative led by the IRD (Institut de Recherche pour le Développement) team at the MNHN Paris. In 2008, this work underwent considerable growth with the Sud Expert Plantes project (http://www.sud-expert-plantes.ird.fr/), which enabled the capture of the "Letouzey" database (Radji et al. 2009) of more than 12,500 specimens. Also in 2008, as part of the "African Plants Initiative" (API) project, http://apps.kew.org/herbcat/gotoApi.do, 8,000 images from TOGO Herbarium specimens where captured and published.

DAK on the plants of Togo is relatively large, based on nearly 85% of primary biodiversity data records derived from specimens in the National Herbarium, plus data from other institutions (Ministry of Environment and NGOs etc.) served through biodiversity informatics initiatives such as GBIF. Data on plants of Togo have not yet been integrated and assessed to establish how complete the site inventories are across the country, so that appropriate levels of confidence can be applied; these gaps in knowledge directly affect the fitness-for-use of the data (Otegui et al. 2013).

## Project

**Figure 1.**
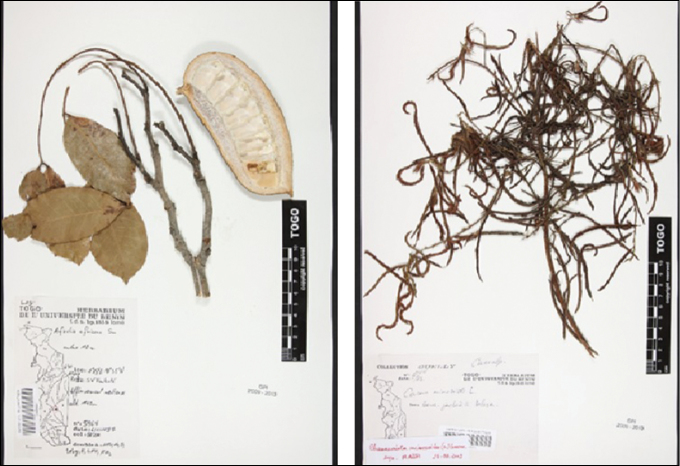
Pictures from the GPI project gallery showing the stamping and the project period.

The database of the National Herbarium of Togo is the result of several digitisation programmes:

– 2003–2006 RIHA Project: initiated by IRD and funded by MAEE France, allows Togo's Herbarium, to learn and become familiar with the RIHA platform. https://www.ird.fr/les-partenariats/renforcement-des-capacites/des-program mes-specifiques/bourses-d-echanges-scientifiques-et-technologi ques-best

– 2008–2011 SEP (Sud Expert Plantes programme (http://www.sud-expert-plantes.ird.fr/ projets/Herbiers_et_J_Botaniques funded by MAEE France (SEP Project N°206) (http://www.sud-expert-plantes.ird.fr/ projets/dossier_206) makes it possible for the University of Lomé to start modernising its herbarium by assembling herbarium specimens to international standards and to capture labels’ data into the RIHA database.

– 2008–2010 API African Plants Initiative Projects, funded by Mellon Foundation through JsTor https://plants.jstor.org/, allows the publishing of the first 8,000 specimens digitalised (Figure 1).

– 2016–2018 BID Project (BID-AF2015-0004-NAC), led by GBIF secretariat and funded by the European Union https://www.gbif.org/project/82693/ strengthening-the-biodiversity-stakeholders-network-in-togo)

## Personnel

Sodjinou Komlan Edjèdu, Allassani Stephane, Tchani Watchiou, Abotsi Eli, Dogbé Yawa and Koda Donko.

## Data published through

https://www.gbif.org/dataset/3294d36a-987c-4dcb-8ecf-bd2082796f08#

## Taxonomic coverage

**Figure 2.**
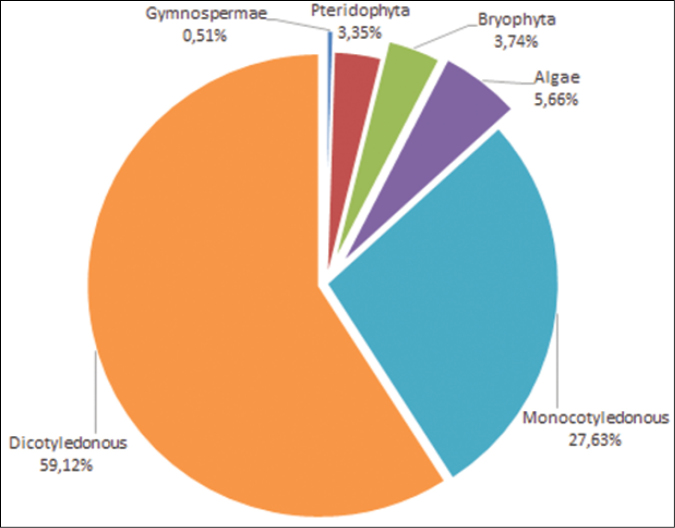
Taxonomic coverage of TOGO dataset in term of specimens.

Most specimens in Togo Herbarium belong to class Magnoliopsida (2,101 specimens) and Liliopsida (8,508 specimens). These classes are followed by Filicopsida (505 specimens), Lycopsida (138 specimens), Coniferopsida (104 specimens), Equisetopsida (24 specimens), Ophioglossopsida (14 specimens), Gnetopsida (6 specimens), Taxopsida (4 specimens), Cycadopsida and Ginkgopsida (both with 2 specimens) and Psilotopsida (1 specimen) (Figure 2).

Togo herbarium contains specimens belonging to 194 families, of which 13.25% of the specimens belong to Leguminosae-Papilionoideae, followed by Graminae (7.73%), Rubiaceae (7.37%), Euphorbiaceae (6.69%), Asteraceae (5%), Cyperaceae (4.50%), Gleicheniaceae (2.66%), Leguminosae-Caesalpinioideae (2.24%), Combretaceae (2.12%), Commelinaceae (1.90%), Moraceae (1.74%), Scrophulariaceae (1.72%), Ulmaceae (1.66%), Leguminosae-Mimosoideae (1.63%), Asclepiadaceae and Apocynaceae (1.57%).

**Figure 3.**
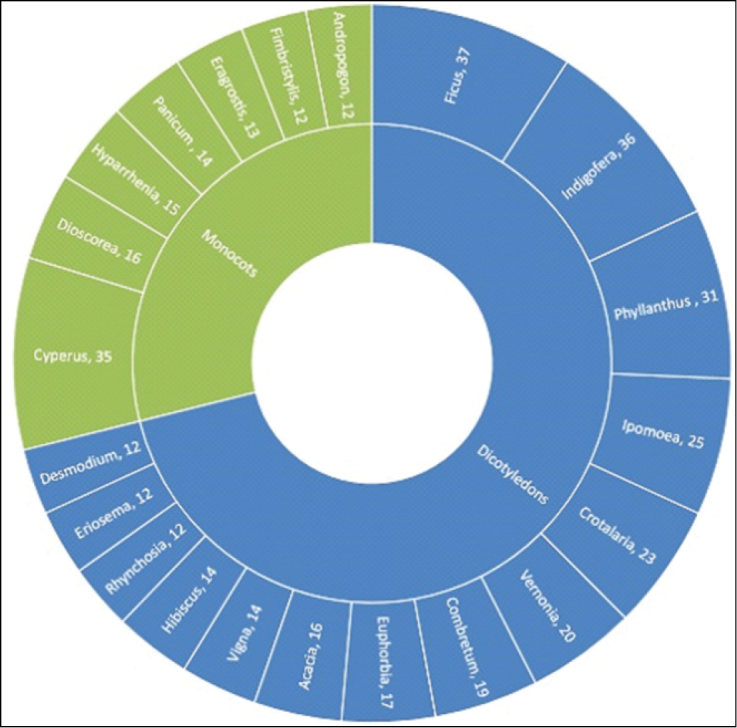
Number of species above 10 per genus in major taxonomic groups.

The herbarium includes 198 genera, significant ones amongst them being *Phyllanthus* (344 specimens), *Ficus* (207 specimens), *Indigofera* (193 specimens), *Combretum* (165 specimens), *Crotalaria* (148 specimens), *Vernonia* (119 specimens), *Euphorbia* (92 specimens), *Trema* (92 specimens), *Cyperus* (168 specimens), *Eriosema* (82 specimens), *Fimbristylis* (82 specimens) and *Uvaria* (45 specimens). Although the herbarium contains specimens from all major botanical groups, Phanerogams (angiosperms-gymnosperms), Pteridophyta (ferns), Bryophyta and Thallophyta (algae-lichens-fungi), the dataset used for this paper covers exclusively Angiosperms (Dicotyledonous and Monocotyledonous) (Figure 3).

The collection includes 508 singletons from 117 different families that represent 4% of the total record. Important families concerned are Graminae (43 specimens), Leguminosae-Papilionoideae 35, Orchidaceae 26, Rubiaceae 25, Euphorbiaceae 22 and Cyperaceae 21 species. Major genera are *Ficus* and *Indigofera* (9 specimens), *Dioscorea* (8 specimens), *Eulophia* (7 specimens), *Crotalaria* and *Phyllanthus* (6 specimens) and *Ceropegia* (5 specimens).

## Taxonomic ranks

**Kingdom**: Plantae

**Division**: Magnoliophyta (Cronquist, Takht. & W. Zimm., 1996)

**Phylum**: *Spermaphyta*

**Class**: *Magnoliopsida, Liliopsida*

**Family**: Agavaceae, Alismataceae, Amaryllidaceae, Anthericaceae, Araceae, Asparagaceae, Aspidiaceae, Cannaceae, Commelinaceae, Costaceae, Cyperaceae, Dioscoreaceae, Dracaenaceae, Droseraceae, Eriocaulaceae, Erythroxylaceae, Gramineae, Hyacinthaceae, Hydrocharitaceae, Hydrophyllaceae, Hypoxidaceae, Iridaceae, Lemnaceae, Liliaceae, Linaceae, Marantaceae, Musaceae, Najadaceae, Opiliaceae, Orchidaceae, Palmae, Pandanaceae, Pontederiaceae, Smilacaceae, Taccaceae, Typhaceae, Xyridaceae, Zingiberaceae.

Acanthaceae, Aizoaceae, Amaranthaceae, Anacardiaceae, Annonaceae, Apocynaceae, Araliaceae, Aristolochiaceae, Asclepiadaceae, Avicenniaceae, Azollaceae, Balanitaceae, Balanophoraceae, Balsaminaceae, Basellaceae, Begoniaceae, Bignoniaceae, Bixaceae, Bombacaceae, Boraginaceae, Burseraceae, Cactaceae, Campanulaceae, Capparaceae, Caricaceae, Caryophyllaceae, Casuarinaceae, Cecropiaceae, Celastraceae, Ceratophyllaceae, Chenopodiaceae, Chrysobalanaceae, Cochlospermaceae, Colchicaceae, Combretaceae, Compositae, Connaraceae, Convolvulaceae, Crassulaceae, Cruciferae, Cucurbitaceae, Dichapetalaceae, Dilleniaceae, Dipterocarpaceae, Ebenaceae, Euphorbiaceae, Flacourtiaceae, Flagellariaceae, Gentianaceae, Gesneriaceae, Goodeniaceae, Guttiferae, Haloragaceae, Hernandiaceae, Hippocrateaceae, Icacinaceae, Irvingiaceae, Labiatae, Lauraceae, Lecythidaceae, Leeaceae, Leguminosae, Leguminosae-Caesalpinioideae, Leguminosae-Mimosoideae, Leguminosae-Papilionoideae, Lentibulariaceae, Loganiaceae, Loranthaceae, Lycopodiaceae, Lythraceae, Malpighiaceae, Malvaceae, Melastomataceae, Meliaceae, Menispermaceae, Molluginaceae, Moraceae, Moringaceae, Myristicaceae, Myrsinaceae, Myrtaceae, Nyctaginaceae, Nymphaeaceae, Ochnaceae, Olacaceae, Oleaceae, Onagraceae, Opiliaceae, Oxalidaceae, Pandaceae, Papaveraceae, Passifloraceae, Pedaliaceae, Phytolaccaceae, Piperaceae, Pittosporaceae, Plumbaginaceae, Podostemaceae, Polygalaceae, Polygonaceae, Portulacaceae, Proteaceae, Punicaceae, Ranunculaceae, Rhamnaceae, Rhizophoraceae, Rosaceae, Rubiaceae, Rutaceae, Salicaceae, Santalaceae, Sapindaceae, Sapotaceae, Saxifragaceae, Scrophulariaceae, Simaroubaceae, Solanaceae, Sphenocleaceae, Sterculiaceae, Thymelaeaceae, Tiliaceae, Turneraceae, Ulmaceae, Umbelliferae, Urticaceae, Verbenaceae, Violaceae, Vitaceae, Zygophyllaceae.

## Spatial coverage

**Figure 4.**
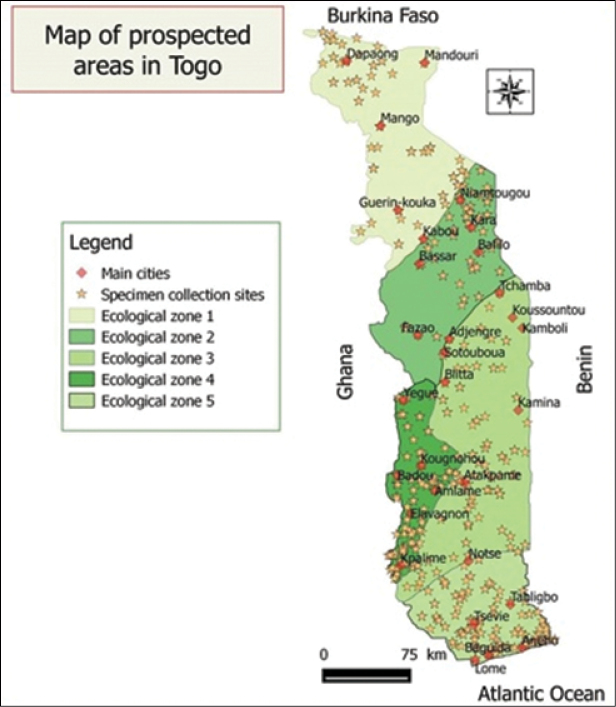
Map of prospected areas in the whole country.

The described dataset collections come from all over Togo. Togo is located in West Africa which has an area of 56,600 km^2^. It stretches for 600 km from North to South and East to West between 50 and 150 km wide (Figure 4).

Togo Herbarium specimens were collected through the 5 Togo ecological zones (ZE) (Figure 9) recognised by Ern (1979). ZE.I refers to the Northern Plains Savannah, with Sudan savannah as the predominant vegetation with few islands of dry forests and gallery forests. ZE.II is covered with a mosaic of dry forests of mountain and forest galleries and climate is Sudano-Guinean. ZE.III corresponds to the Guinean savannahs of central area plains enjoying a tropical climate with one rainy season. Semi-deciduous forests are noticed in the southern part and dry forests in the northern part. ZE.IV covers the southern part of the Togo Mountains and has a sub-equatorial transition climate. The vegetation is constituted of rainforests, on deep red lateritic soils. This ZE is the domain of dense semi-deciduous forests. The last one, ZE.V is a coastal plain of southern Togo with a subequatorial climate marked by a deficit in rainfall. It is characterised by a climate with two rainy seasons and the vegetation is set up by a mosaic of savannah, farmland and dry forests (Kokou and Caballé 2000).

## Coordinates

Togo is located between 6° and 11° latitude and 1° East longitude (Figure 4). Data in Togo National Herbarium are referenced between 11.05 latitude and 0.2 and 1.85 longitude. Data are also from "Mont Agou" that is the highest peak in Togo, at an altitude of 986 m and located between Amoussoukopé and Kpalimé in ecological zone IV.

## Temporal coverage

**Figure 5.**
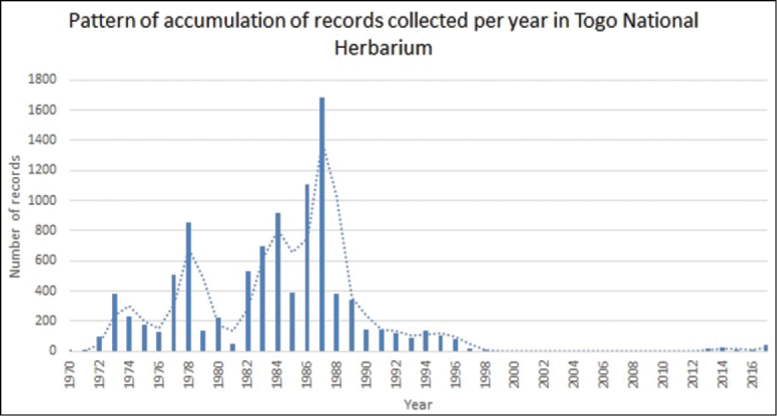
Pattern of accumulation of records collected per year in Togo National Herbarium.

**Figure 6.**
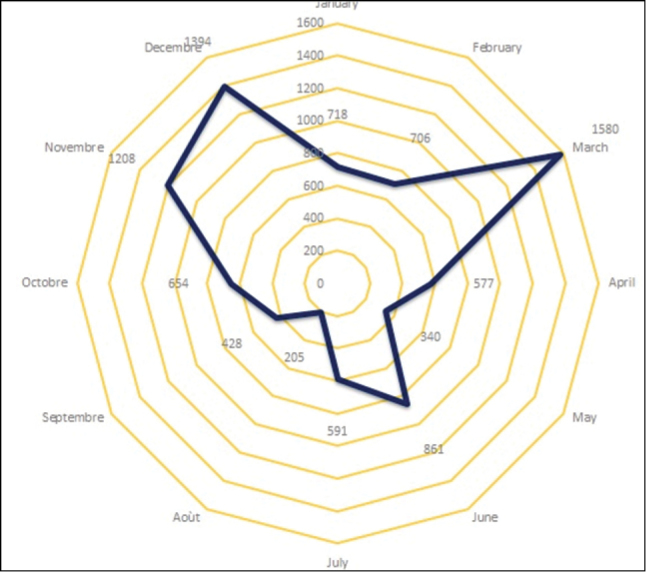
Pattern of record accumulation of Togolese plants collected per month.

With respect to the temporal coverage of the specimens, 5,895 (47.16%) consist of specimens collected from 1970 to 1984, most of them used for the botanical species description and the drafting of the Togo's Flora (Brunel 1975, 1987; Brunel et al. 1984). Newer specimens came from the fieldwork of local researchers (Kokou and Caballé 2000, Akpagana and Guelly 1994, Akpagana 1992b), teaching trips from teachers with students in Plant Biology and the Environment Department from the University of Benin (University of Lomé from 2001 onwards). These collections represent 60% of the current holdings (Figures 5, 6).

## Natural collections description

**Figure 7.**
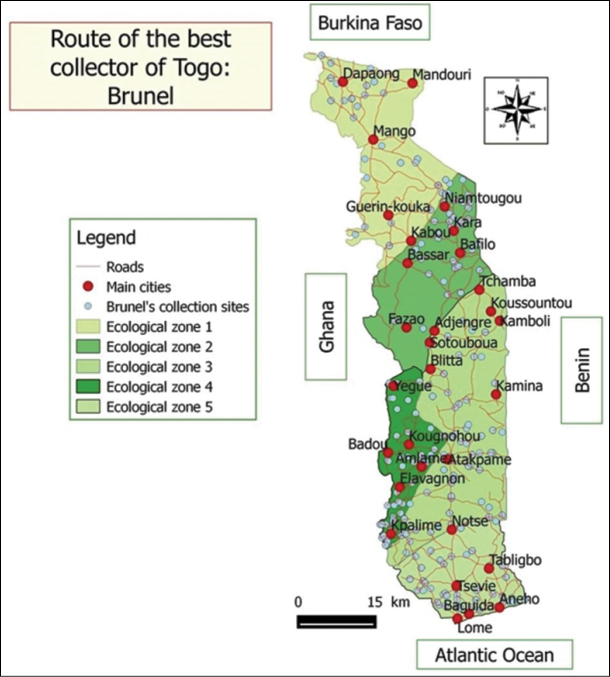
Route of the best collector in Togo: Brunel.

**Figure 8.**
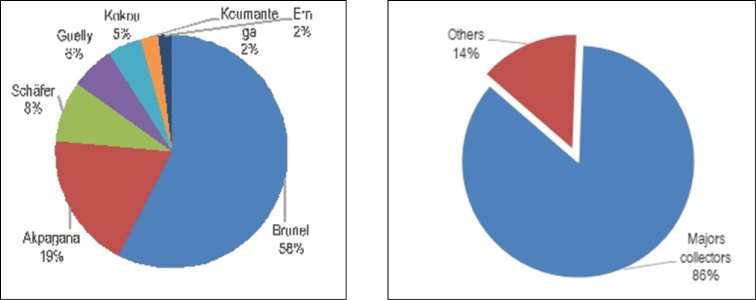
Major collectors who collected around Togo’s ecological area from 1971 to 2017 and comparison to the rest of the collection.

The herbarium specimens were mainly collected in Togo (99%). The remaining 1% come from neighbouring countries such as Ghana, Benin and Côte d'Ivoire collected by Togolese researchers on research trips. These specimens date from the second half of the 20th century. Today, the Herbarium occupies an area of approximately 100 m^2^ and is located on the ground floor of the building that houses the Dean’s Office of the Faculty of Sciences of the University of Lomé. Thanks to SEP Project N°206 --funded in 2008 by the French Ministry of Foreign Affairs, the storage for 12,572 specimens was upgraded to international standards by use of new closed door wooden cabinets. The collection is arranged in alphabetical order by Family, Genus and Species names. There are no type specimens kept in the Herbarium but the only recognised endemic species is well-represented by 16 specimens. The main collectors are: Brunel (6227 specimens, Figures 7 and 8), Akpagana (2023), Schäfer (911), Guelly (654), Kokou (508), Koumantega (276) and Ern (200) (Figure 8). While most of the samples have little useful information, those of Schäfer PA are well georeferenced and contain an unsuspected amount of information about traditional knowledge as well as the vernacular names of the samples in its collection (Figure 8).

## Collection name

University of Lomé herbarium: *Herbarium togoense*; Index Herbariorum Acronym: TOGO.

## Methods

The compilation of this dataset was based on digitised data and experiences gained from the RIHA and SEP projects, as well as an updated inventory of existing resources (collections and contacts). In particular, it relied on the establishment of databases in Herbaria (Gaston 2000; Bebber et al. 2010), the standardisation and homogenisation of data through the reference systems (names of plants, localities and people). Consultation of the renowned botanical databases has been carried out (http://www.eol.org; https://www.biodiversitylibrary.org). Linkage with forest and ecological databases (inventories) has been undertaken, as well as on-line consultation of data via relevant international portals such as GBIF (http://www.gbif.org). During the fieldwork, the species were recorded as presence / absence before being sampled for the herbarium - both in surveys of inventories (transects), forest ecology (setting up plots) and ethnobotany. Species identification was made with Togo's flora (Brunel et al. 1984), Senegal's flora (Berhaut 1967), Senegal Illustrated Flora (Berhaut 1971–1988), Descriptive Flora of Côte d'Ivoire (Ake Assi 1984) and Forest Flora of Côte d'Ivoire (Aubreville 1959). Further information was collected from Flora of West Tropical Africa (Hutchinson and Dalziel 1954–1972) and from the Enumeration of Tropical African Flowering Plants (Lebrun and Stork (1991–1992, 2006, 2008, 2018). The nomenclature used is that of the original authors and classification in the database released in this paper follows Hutchison and Dalziel (1953–1967) as currently maintained in the Togo National Herbarium. The International Plant Names Index (http://www.ipni.org) and Botanist's Index (http://kiki.huh.harvard.edu/databases/botanist_index.html) have been used. The list of synonyms followed The Plant List www.theplantlist.org. For Lycophytes and Ferns, the classification followed that established by Christenhusz et al. (2011), while that of flowering plants followed Cronquist (1981).

### Method step description

The fieldwork and herbarium specimens study followed traditional procedures (Mori et al. 2011).

**Data processing.** Details -measures, label facts, nomenclature etc. from all studied specimens were entered into a MS-Access database (RIHA database) designed and built for that purpose. Additional tables and queries were created to record specimen measures as well as bibliographic, taxonomic and nomenclatural information - these are not included in this dataset, but nomenclature information is available through 'The Plant List' (2013) and 'African Plant Database (2018). Specific guidelines regarding data entry and quality control procedures followed Pando et al. (1999). The dataset was published in a standardised format using the Integrated Publishing Toolkit (Robertson et al. 2014) hosted by GBIF France. The standardised format is Darwin Core Archive (DwC-A, GBIF 2010), which is a biodiversity data standard that makes use of Darwin Core terms (Wieczorek et al. 2012). A Darwin Core Archive is a zip file containing a data file in tab delimited text (.txt) format, an xml file describing the data file, the relationships between the archive’s data files when there is more than one (meta.xml) and a machine readable dataset metadata in XML format (eml.xml), complying with GBIF Metadata Profile (GBIF 2011), based on EML.

**Study extent description**: The analysis of this dataset reveals that eco-floristic zone IV is the most significant and prospected with 31.63% of the herbarium specimens whereas ecological zone II remains the least prospected with 11.33% of the collected specimens.

Although quite rich, Togolese spontaneous flora remains incompletely known (Akpagana 1992b, Radji 1997, Kokou 1998).

**Figure 9.**
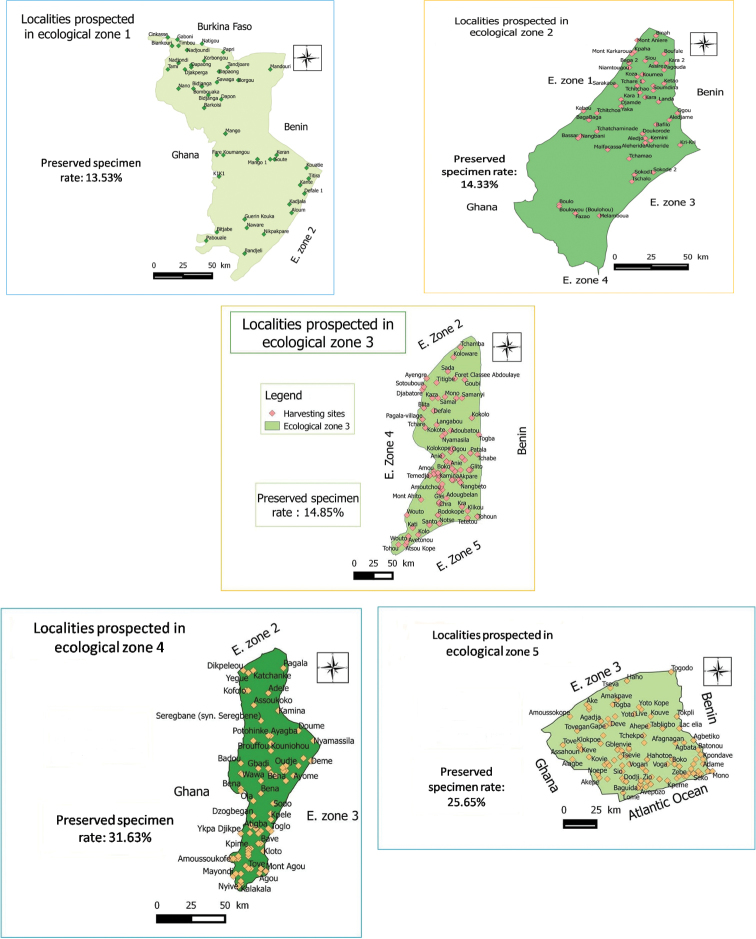
Localities botanically prospected by ecological zone in Togo.

Figure 9: Localities botanically prospected by ecological zone in Togo

**Sampling description**: The specimens deposited in the TOGO Herbarium come from several research projects mainly involving first lecturers in Botany in the university followed by the first PhD students studying in French universities whose fieldwork was carried out in Togo. As a result, specimens were collected under a variety of methods and diverse objectives. The situation is the same with materials coming from donations or purchases (e.g. herbarium of Ern). When possible, duplicates of specimens have been sent to renowned herbaria. Some duplicates of herbarium specimens (5,403, 36.02% of the collection) from Togo were deposited in other herbaria committed to long-term maintenance. These where: B, BR, IFAN, K, LMU, MO, MPU, P, STR, UK, US and WAG (http://sweetgum.nybg.org/science/ih/.

During the SEP project (2008–2012), 6 students studying for Master's degrees in Plant Biology were recruited to assemble herbarium specimens deposited by collectors in presses and sometimes in Canson papers. Most specimens have original collectors’ labels. Trained in the use of RIHA database (under Microsoft Access), the students digitised the data as and when the specimens were mounted. Data were exported to Excel for publication..

**Quality control description**: The present dataset was updated to match the Cronquist classification for the orders and families of flowering plants and all species names were checked for validity (spelling, synomyms and authorship) against online databases: http://www.ipni.org/ipni/plantnamesearchpage.do; http://www.theplantlist.org/; http://www.ville-ge.ch/musinfo/bd/cjb/africa/recherche.php.

## Datasets

### Dataset description

**Object name**: Togo National Herbarium database

**Character encoding**: UTF-8

**Format name**: Darwin Core Archive format

**Format version**: 1.0

**Distribution**: http://ipt-togo.gbif.fr/resource?r=herbarium_database&v=1.5

**Publication date of data**: 2018-01-05

**Language**: French

**Licences of use**: This database “Togo National Herbarium database” is made available under licence Creative Commons Attribution (CC-BY) 4.0 License

**Metadata language**: English

**Date of metadata creation**: 2018-01-05

**Hierarchy level**: Dataset
